# What Causes Carbon-Centered Radicals to Pyramidalize? It Depends on the Type of Radical

**DOI:** 10.1021/acs.joc.5c02141

**Published:** 2026-01-05

**Authors:** Gary W. Breton

**Affiliations:** Department of Chemistry and Biochemistry, 5659 Berry College, Mount Berry, Georgia 30149, United States

## Abstract

Carbon-centered radicals, with a single electron on the carbon atom, could potentially adopt a planar geometry similar to that of alkyl carbocations, a tetrahedral geometry similar to that of alkyl carbanions, or a geometry somewhere in between. Simple alkyl radicals are known to adopt a slightly pyramidalized geometry due to a combination of torsional and hyperconjugative interactions. Which of these interactions is primarily responsible for determining the final adopted geometries has, up until now, remained unknown. Harnessing the power of natural bond orbital calculations to perform geometry optimizations with specific donor–acceptor interactions deleted, it is now demonstrated that hyperconjugative interactions primarily dictate radical geometries. For simple alkyl radicals, negative hyperconjugative interactions are most important, while for bridgehead radicals, positive hyperconjugative interactions are most important.

## Graphical abstract

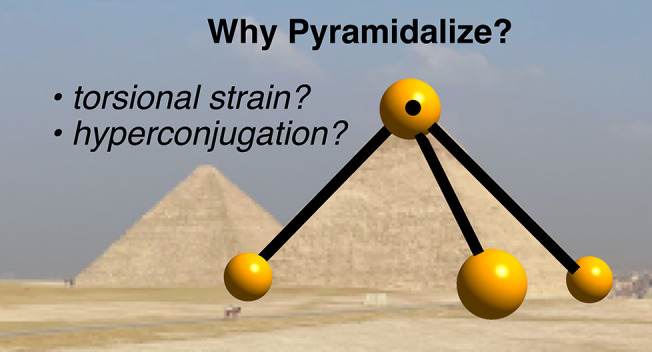

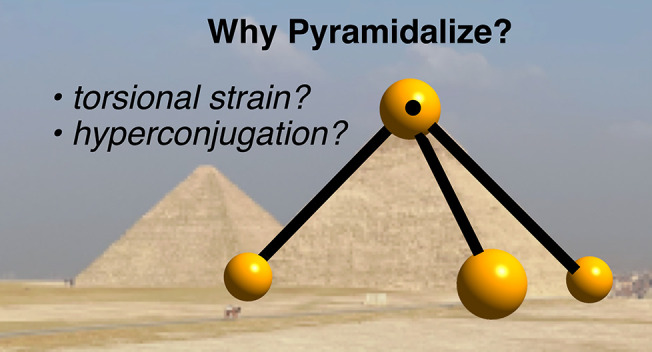

## Introduction

Carbon-centered radicals are frequently encountered intermediates in organic chemistry.^1,2^ Much experimental and theoretical work has been directed toward understanding the effects of structure and substitution on the stability of the radical center.^1–3^ Much less work, however, has been directed toward understanding the reasons why these radicals adopt particular geometries.^4–13^ Radicals find themselves on the spectrum between planar carbocations that lack any electrons in the p-orbital of the carbon atom and tetrahedral carbanions that have two electrons in an sp^3^-hybridized orbital at the carbon. With a single electron at the carbon atom, the radical carbon could potentially adopt a planar geometry, a tetrahedral geometry, or a geometry somewhere in between (i.e., pyramidalized).

It is well-established that the simplest carbon-centered radical, the methyl radical, is planar.^1,9^ It is also known that upon sequential substitution of the hydrogen atoms of the methyl radical with alkyl groups (e.g., methyl), increasing pyramidalization of the radical-bearing carbon atom is observed to occur.^4–7^ Early calculations, using the unresticted Hartree Fock (UHF) method, were compiled by Paddon-Row and Houk in their 1981 paper that directly addressed the “origin” of the pyramidalization of the *tert*-butyl radical.^7^ They measured the extent of pyramidalization of the radical carbon by determining the angle of deflection of one of the C–C bonds from the plane defined by the central (radical) carbon and the other two atoms to which it was attached. For the methyl (C_*3V*_), ethyl (C_*s*_), 2-propyl (C_*s*_), and *tert*-butyl (C_*3V*_) radicals, they reported angles of 0.0°, 6.2°, 15.9°, and 22.1°, respectively. In an earlier series of papers, Pacansky and co-workers suggested that these radicals adopt pyramidalized geometries due to repulsive torsional interactions between the CH bonds of the methyl groups and vicinal CH and/or CC bonds, as well as the radical center’s singly occupied molecular orbital (SOMO).^4–6^ They postulated that the partially filled SOMO could exert a torsional influence akin to a “lone pair or bond.”^6^ Upon increasing substitution of the CH bonds of the methyl radical with methyl groups, additional pyramidalization at the carbon is required to offset the increased torsional interactions.^6^ Paddon-Row and Houk acknowledged the important contribution of the torsional effects but further suggested that hyperconjugative interactions of the unpaired electron in the SOMO with properly aligned CH bonds of the attached methyl groups were also relevant.^7^ Indeed, it was observed that hyperconjugation results in specific lengthening of one of the CH bonds (1.091 Å) relative to the other two (1.084 Å) for the *C*_*3V*_-symmetric *tert*-butyl radical.^6,7^ Interestingly, these authors briefly discussed in footnotes of one of their papers that the competing torsional and hyperconjugative effects create a “chicken-and-egg indeterminancy” as to which is ultimately responsible for the radical adopting a specific geometry.^7^ They noted that any geometry changes adopted by the radical in an effort to minimize torsional interactions will inevitably result in increased stabilizing hyperconjugative interactions and vice versa.

The significance of hyperconjugation within carbon-based radicals has since been confirmed and used to help explain trends in homolytic bond strengths and to interpret EPR spectra.^14,15^ In 2012, Sokolov reinvestigated the *tert*-butyl radical at a much higher computational level (CCSDT­(Q)).^12^ Sokolov confirmed the C_*3V*_ minimum for the *tert*-butyl radical with a pyramidalized carbon (22.9° angle, as defined above). He also noted the elongation of the CH bonds of the methyl groups that were aligned with the SOMO versus those that were not (i.e., 1.101 Å vs 1.092 Å). Natural bond orbital (NBO) calculations supported the idea that hyperconjugative interactions of the SOMO with the aligned antibonding σ*_CH_ orbitals were responsible for the elongation. Sokolov termed this “negative hyperconjugation” because it was opposite in sense to the more conventionally observed hyperconjugation, in which σ bonds delocalize electron density into aligned electron-deficient orbitals (e.g., the p-orbitals of carbocations).^12,16^ Note that negative hyperconjugation also serves to delocalize radical spin character from the radical carbon onto the adjoining groups. Interestingly, however, Sokolov did not speculate upon the involvement of conventional “positive hyperconjugation,” in which the aligned σ_CH_ orbitals could donate electron density into the SOMO. Such hyperconjugation could be important because the SOMO is electron-deficient because it only houses a single electron. Positive hyperconjugation would also be expected to contribute to bond elongation of aligned CH bonds due to depletion of the electron density within the interacting σ bond. Indeed, earlier work has recently discovered that consideration of both negative and positive hyperconjugation is important for properly rationalizing the relative energies of substituted bridgehead radicals.^17^

The separation of hyperconjugation into positive and negative effects allows for a more nuanced evaluation of the interactions.^17^ Furthermore, NBO calculations can prove to be especially powerful and insightful when geometry optimizations are carried out with deletions of specific donor–acceptor interactions, thereby revealing the preferred geometry in the absence of such interactions.^18^ Indeed, Ingold and DiLabio briefly related in a footnote within a paper that geometry optimization of the ethyl radical when hyperconjugation interactions were deleted resulted in a lengthening of the C–C bond length.^15a^ Unfortunately, no additional information about geometric changes was provided. This raised the question as to whether NBO optimization calculations on the simple carbon-centered radicals with deletions of targeted donor/acceptor channels might allow for the determination of which type of interaction—torsional or hyperconjugative—had the greater influence on radical geometries, thereby resolving the “chicken-and-egg” problem raised earlier by Paddon-Row and Houk.^7^

## Results and Discussion

### Methyl Radical

Optimization of the methyl radical (UωB97X-D/6-311++G­(d,p)) leads to a completely planar geometry of C_*3V*_ symmetry (Figure 1). The CH bonds have lengths of 1.081 Å. NBO calculations revealed that the unpaired electron resides in a p-orbital of the carbon atom. Any type of hyperconjugation is prohibited for this molecule because of the orthogonality of the SOMO and CH σ and σ* orbitals. The NBO spin population on the carbon atom is 1.090 *e*, and that on the three hydrogen atoms is −0.03 *e*. This limited amount of spin transfer is attributed to the phenomenon of spin polarization, a process by which the spin of the opposite sense is localized on neighboring atoms due to correlation effects between the unpaired electron and the electron pair in the bond between the two atoms.^15^

**Figure 1:**
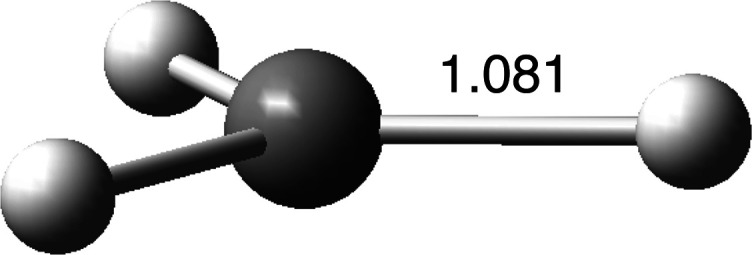
Select data for the optimized geometry of the methyl radical. Bond lengths are given in Å.

### Ethyl Radical

Geometry optimization of the ethyl radical (C_*S*_ symmetry) affords a slightly pyramidalized geometry about the radical carbon (Figure 2 and Table 1). As mentioned earlier, the extent of pyramidalization of radical carbons has been measured previously by the angle of deflection of one of the C–C bonds to the radical carbon relative to a plane defined by the radical carbon and the other two atoms to which it is attached.^7^ Since this manner of measurement can sometimes be ambiguous, pyramidalization was determined more directly by measuring the distance, *d* (in Å), of the radical carbon from the plane defined by the three atoms to which it is attached, as illustrated in Figure 2A. This is especially helpful for the bridgehead radicals discussed later. For the ethyl radical, this distance is 0.050 Å, which corresponds to a 4.6° bending angle in qualitative agreement with the 6.2° bending angle referenced earlier at a lower level of calculation.^7^ For reference, one of the carbons of fully saturated ethane (optimized under identical conditions) is 0.391 Å from the plane defined by the three atoms to which it is attached (in this case, a carbon atom and two hydrogen atoms). Thus, the radical carbon of the ethyl radical is approximately 13% pyramidalized relative to one of the carbons of ethane. The two CH bonds directly attached to the radical carbon have lengths (1.083 Å) nearly identical to those of the methyl radical. The C–C bond length of 1.485 Å is shortened relative to a typical C–C single bond (1.54 Å) because of the sp^2^-like nature of the radical carbon. As has been noted before, the CH of the methyl group that is aligned with the SOMO is of greater length (1.101 Å) relative to the other two CH bonds (1.094 Å).^4,7^ The reason for the selective bond lengthening is provided by NBO analysis, which shows both positive (i.e., σ_CH_ → SOMO) and negative (SOMO → σ*_CH_) hyperconjugation, both of which would weaken this particular CH bond and lead to its modest lengthening (Figure 3). The energy contributions from these two hyperconjugation mechanisms are similar, with the positive hyperconjugation being somewhat more impactful based on second-order perturbation energies (Table 1, columns 4 and 5). The spin delocalizing effect of the negative hyperconjugation results in depletion of the spin population at the radical carbon atom (1.018 *e*) relative to that of the methyl radical (Table 1, column 3).

**Figure 2:**
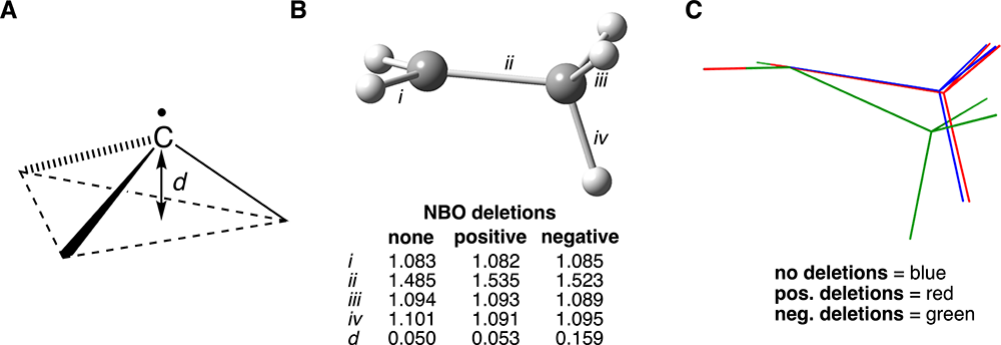
(A) Illustration that defines distance “*d*” (in Å) as a measure of radical carbon pyramidalization. (B) Select data for the optimized geometries of the ethyl radical without (as shown) and with hyperconjugation deletions. Bond lengths in Å. *d* = distance (Å) of radical carbon from the plane defined by the three atoms to which it is attached. (C) Visualization of geometry change resulting from no deletions (blue), positive hyperconjugation deletion (red), and negative hyperconjugation deletions (green). The radical carbon and two hydrogens of the CH bonds for the three structures are intentionally superimposed.

**Table 1:** Properties of the Ethyl Radical from NBO Calculations on Optimized Geometries Following Specified Hyperconjugation Deletions

NBO deletion^a^	Δ*E* ^b^ (kcal/mol)	spin population^c^ (*e*)	positive hyper^d^ (kcal/mol)	negative hyper^e^ (kcal/mol)	total hyper^f^ (kcal/mol)
none^g^	0.0	1.018	12.90	10.93	23.83
positive^h^	+0.91	1.034	10.18	9.45	19.63
negative^i^	+1.65	1.025	12.82	6.73	19.55

a Indicates whether positive or negative hyperconjugation channels were deleted during geometry optimization.

b The energy of the resulting molecule relative to the optimized geometry with no deletions.

c The natural spin population as derived from the NBO calculation on that geometry.

d The total of all major positive hyperconjugation mechanism energy contributions for that geometry.

e The total of all major negative hyperconjugation mechanism energy contributions for that geometry.

f The total of both hyperconjugation mechanism energy contributions for that geometry.

g Geometry optimization of the radical with no NBO deletions.

h Geometry optimization of the radical with NBO deletions of major positive hyperconjugation mechanism contributions.

i Geometry optimization of the radical with NBO deletions of major negative hyperconjugation mechanism contributions.

**Figure 3:**
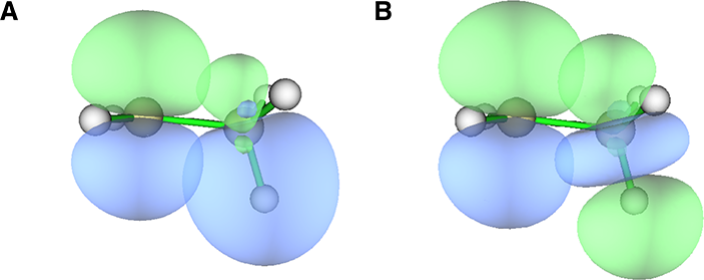
NBO orbitals for the ethyl radical depicting (A) positive hyperconjugation and (B) negative hyperconjugation interactions of the radical carbon SOMO (toward the left) with the properly aligned CH σ and σ* orbitals (respectively) of the methyl group.

For all the simple alkyl radicals, the primary hyperconjugative interactions (both positive and negative) are between the SOMO and the neighboring CH bonds of the methyl group(s), such as those shown in Figure 3 (see Figure S1 in the Supporting Information for individual values). Thus, the geometry of the ethyl radical was next reoptimized but with selective deletion of the major positive hyperconjugative interactions only. Only a slight increase in the extent of pyramidalization resulted (*d* = 0.053 Å) relative to that of the parent ethyl radical (*d* = 0.050 Å). The energy of the reoptimized geometry increased by just 0.91 kcal/mol. Recalculating the NBO interactions at this geometry revealed that both the positive and negative hyperconjugation energy contributions dropped slightly as a result of the change in geometry. The spin population on the radical carbon increased (1.034 *e*). While the lengths of the two CH bonds directly attached to the radical carbon were essentially unaffected, the length of the aligned methyl CH bond became shorter, such that the two different types of CH bonds of the methyl group became nearly identical (1.091 and 1.093 Å). Note that the C–C bond was lengthened from 1.485 to 1.535 Å. The rather subtle change in overall geometry from the original geometry is more readily revealed by Figure 2C, in which the geometry resulting from positive hyperconjugation deletion (in red) is superimposed onto the original geometry (in blue) with intentional alignment of the radical carbon and the two hydrogen atoms of the directly connected CH bonds.

The original geometry of the ethyl radical was again reoptimized, but this time with selective deletions of the negative hyperconjugative interactions. The result was a significant increase in the extent of pyramidalization at the radical carbon (*d* = 0.159 Å), which places it at 41% of the pyramidalization of one of the carbons of ethane. This is clearly illustrated by Figure 2C for the structure in green relative to the other two structures. There was a significant drop in stabilizing interactions by the negative mechanism but only a slight decrease by the positive mechanism (Table 1). The spin population at the radical carbon was again observed to increase (1.025 *e*). Similar to what had been observed upon deletion of the positive hyperconjugation channels, the C–C bond length increased to 1.523 Å, in qualitative agreement with Ingold’s observation mentioned above.^15a^ Also, the two types of CH bonds of the methyl group both shortened in length (1.095 Å for one CH versus 1.089 Å for the remaining two). The directly attached CH bonds were again hardly affected. The observations from the ethyl radical are next compared to the results of similar calculations on the 2-propyl and *tert*-butyl radicals.

### 2-Propyl Radical

Optimization of the 2-propyl radical was carried out with C_*S*_ symmetry (Figure 4 and Table 2). As has been noted before, the radical carbon saw increased pyramidalization relative to the ethyl radical with the addition of methyl substitution (*d* = 0.087 vs 0.050 Å, respectively).^5^ The radical carbon, therefore, is at 20% of the pyramidalization of the secondary carbon of propane (*d* = 0.426 Å). Again, the CH bonds of the methyl groups that are aligned with the SOMO are of greater length (1.102 Å) relative to the other two (1.097 Å). The total hyperconjugative interactions were somewhat greater than double that of the ethyl radical, and the spin population on the radical carbon decreased to 0.951 *e.*

**Figure 4:**
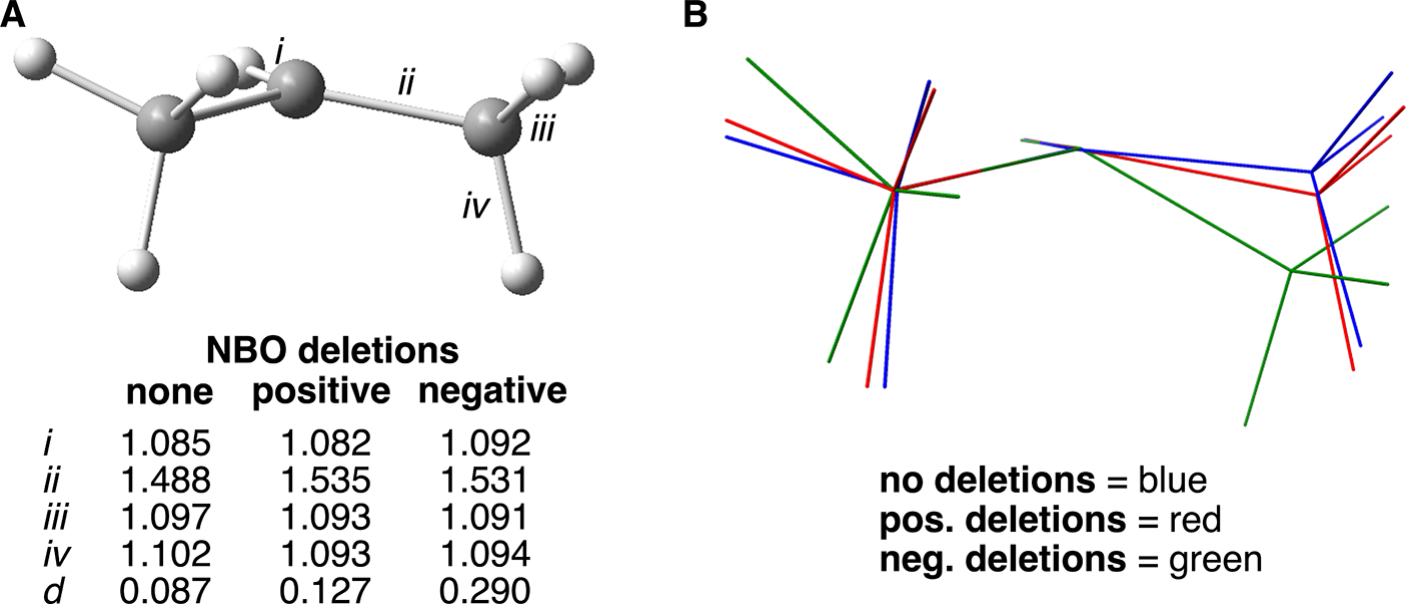
(A) Select data for the optimized geometries of the 2-propyl radical without (as shown) and with hyperconjugation deletions. Bond lengths in Å. *d* = distance (Å) of radical carbon from the plane defined by the three atoms to which it is attached. (B) Visualization of geometry change resulting from no deletions (blue), positive hyperconjugation deletion (red), and negative hyperconjugation deletions (green). The radical carbon, the hydrogen atom of the CH bond, and the carbon of one of the methyl groups are intentionally superimposed.

**Table 2:** Properties of the 2-Propyl Radical from NBO Calculations on Optimized Geometries Following Specified Hyperconjugation Deletions^a^

NBO deletion	ΔE (kcal/mol)	spin population (*e*)	positive hyper (kcal/mol)	negative hyper (kcal/mol)	total hyper (kcal/mol)
none	0.0	0.951	24.58	23.98	48.56
positive	+1.68	0.980	19.80	19.91	39.71
negative	+3.71	0.969	23.71	12.63	36.34

a See footnotes in Table 1 for explanations of terms.

Reoptimization with deletion of the positive hyperconjugation mechanism channels resulted in some further pyramidalization of the radical carbon (*d* = 0.127 Å) and an increase in spin population at the radical carbon (0.980 *e*). Both the positive and negative hyperconjugation energies decreased. As with the ethyl radical, the C–C bonds increased in length (from 1.488 to 1.535 Å), while all three CH bonds of the methyl groups became identical in length (1.093 Å). The directly attached CH bond remained essentially unaffected. Ultimately, however, the change in overall geometry was again subtle, as seen in Figure 4B.

Reoptimization with deletion of the negative hyperconjugation channels again resulted in significant pyramidalization of the radical carbon (*d* = 0.290 Å), as had been observed for the ethyl radical (Figure 4A,B). Thus, this radical carbon has adopted 68% of the pyramidalization observed for the secondary carbon atom of propane. Also observed were a significant rise in overall energy (3.71 kcal/mol) and an increase in observed spin population (0.969 *e*). The methyl CH bond lengths were found to be very similar (1.091 vs 1.094 Å). Not surprisingly, the extent of negative hyperconjugation was especially affected by the change in geometry, and a more significant drop in total hyperconjugation energies was observed than when the positive channels were deleted (Table 2).

### *tert*-Butyl Radical

Optimization of the *tert*-butyl radical was carried out with C_*3V*_ symmetry (Figure 5 and Table 3). The 3° radical carbon exhibited the greatest initial pyramidalization (Figure 5A, *d* = 0.177 Å). The 3° carbon of isobutane has *d* = 0.471 Å; thus, the radical carbon is already at 38% pyramidalization. The CH bonds of the methyl groups that were aligned with the SOMO were again of greater length (1.104 Å) relative to the other two (1.094 Å). The spin population on the radical carbon decreased to 0.887 *e* as more spin was delocalized onto the three adjoining methyl groups (Table 3). The *tert*-butyl radical experienced a large total amount of energy contribution from hyperconjugative interactions due to the participation of three separate methyl groups. Note that the stabilizing contributions of the positive hyperconjugative interactions slightly edged out the negative mechanisms (Table 3).

**Figure 5:**
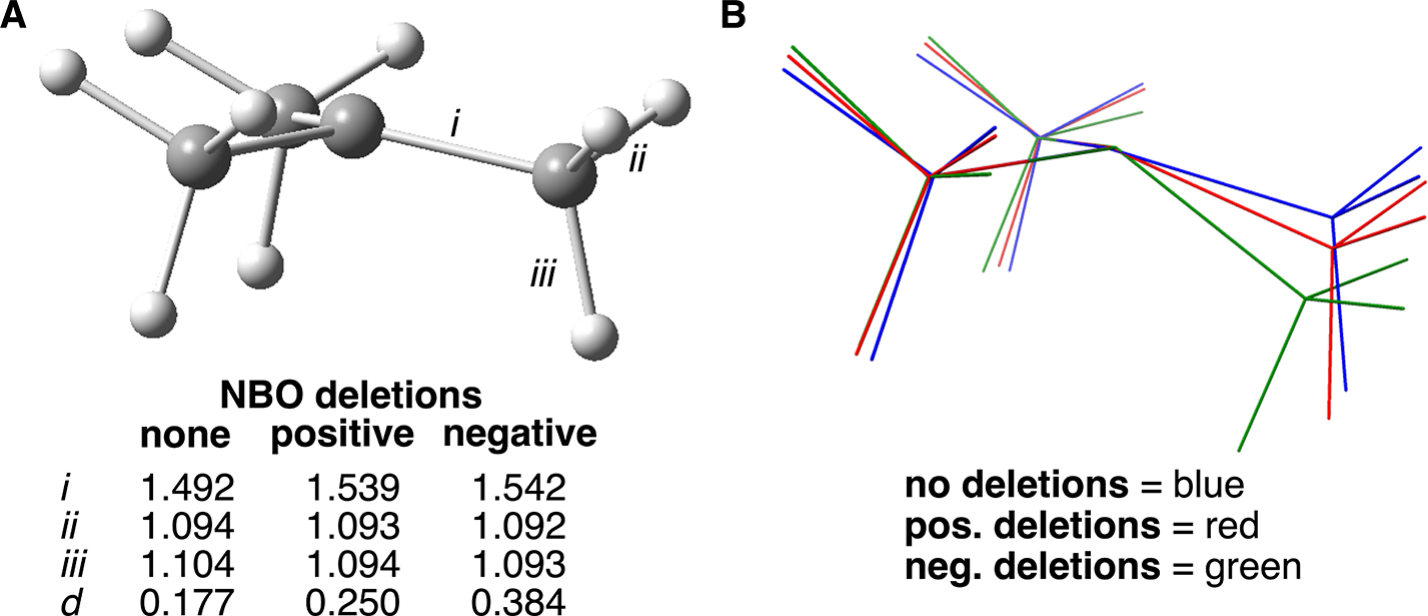
(A) Select data for the optimized geometries of the *tert*-butyl radical without (as shown) and with hyperconjugation deletions. Bond lengths in Å. *d* = distance (Å) of radical carbon from the plane defined by the three atoms to which it is attached. (B) Visualization of geometry change resulting from no deletions (blue), positive hyperconjugation deletion (red), and negative hyperconjugation deletions (green). The radical carbon and two of the methyl group carbons are intentionally superimposed.

**Table 3:** Properties of the *tert*-Butyl Radical from NBO Calculations on Optimized Geometries Following Specified Hyperconjugation Deletions^a^

NBO deletion	ΔE (kcal/mol)	spin population (*e*)	positive hyper (kcal/mol)	negative hyper (kcal/mol)	total hyper (kcal/mol)
none	0.00	0.887	36.75	34.83	71.58
positive	2.55	0.926	29.53	27.71	57.24
negative	4.90	0.923	32.83	19.57	52.40

a See footnotes in Table 1 for explanations of terms.

Deletions of the positive and negative hyperconjugation channels had effects on the *tert*-butyl radical similar to what had been observed earlier for the ethyl and 2-propyl radicals, i.e., an increase in the energy of the system, increased pyramidalization, and increased spin population (Figure 5 and Table 3). The pyramidalization of the radical carbon upon deletion of the negative hyperconjugation channel (*d* = 0.348 Å) amounted to 82% of the corresponding carbon of isobutane. The total hyperconjugation energies decreased with each type of deletion and especially so with the deletion of the negative hyperconjugation channels. Structurally, the C–C bonds increased in length, and the CH bonds of the methyl groups became essentially identical in length (Figure 5A). The superimposed structures of the three optimized geometries (Figure 5B) again highlight the especially significant structural impact upon deletion of the negative hyperconjugation interactions.

### Some Initial Conclusions

Armed with the results of calculations on the methyl, ethyl, 2-propyl, and *tert*-butyl series of alkyl radicals, the following general observations on conventional carbon-centered radicals may be made:

1. As has been reported earlier, both experimentally and computationally, carbon-centered radicals experience increasing pyramidalization with increasing alkyl substitution on the radical carbon.^7–9^ The extent of pyramidalizations reached 13, 20, and 38% of the corresponding carbons in the alkane structures for the ethyl, 2-propyl, and tert-butyl radicals, respectively.
2. Increasing alkyl substitution results in decreased spin population on the radical carbon due to negative hyperconjugation that distributes spin density onto neighboring atoms and groups of atoms.
3. Hyperconjugation, both positive and negative, results in one of the CH bonds of attached methyl groups being lengthened relative to the other two.
4. Generally, optimization of geometries upon deletion of positive hyperconjugation channels resulted in
  a) an increase in energy of the radical,
  b) an increase in the spin population on the radical carbon,
  c) lengthening of the C–C bonds along with equalization of the two types of CH bonds in the connected methyl groups,
  d) a decrease in total hyperconjugation energies, and
  e) *slight* additional pyramidalization of the radical carbon (14, 30, and 53% of the corresponding carbons in the alkane structures for the ethyl, 2-propyl, and *tert*-butyl radicals, respectively).
5. Generally, optimization of geometries upon deletion of negative hyperconjugation channels resulted in (a)–(d), as listed above for deletion of the positive hyperconjugation channels, but significant additional pyramidalization of the radical carbon (41, 68, and 82% of the corresponding carbons in the alkane structures for the ethyl, 2-propyl, and tert-butyl radicals, respectively).

As discussed earlier, the pyramidalization of carbon radicals has been attributed to the avoidance of torsional interactions and maximization of hyperconjugative interactions.^4–7^ Our computational results demonstrate that in the absence of stabilizing hyperconjugative interactions, additional pyramidalization would take place in an apparent effort to further avoid torsional interactions. The reduction of torsional interactions is readily visualized for the *tert*-butyl-based substrates, as shown in Figure 6, as Newman-like projections. Viewing one of the C­(methyl)–C­(radical) bonds of the optimized *tert*-butyl radical in Figure 6a reveals the torsional strain between the methyl CH bonds and the two remaining C–C bonds about the radical carbon. The HCCC dihedral angle is 40.9°. Upon geometry optimization of the *tert*-butyl radical with the negative hyperconjugation channels deleted (see Figure 6B), the dihedral angle opens to 51° with an attending drop in torsional strain. This dihedral angle approaches that of isobutane, with a dihedral angle of 57.8° (Figure 6C). Thus, Pacansky’s earlier suggestion that the unpaired electron exerts its influence much like a bond is not unreasonable.^6^ Therefore, carbon radicals, while sensitive to both hyperconjugative and torsional effects, apparently adopt geometries predominantly in a manner to maximize hyperconjugation, even at the cost of increased torsional interactions. Furthermore, of the two modes of hyperconjugation, positive and negative, it is negative hyperconjugation that is especially pertinent to the geometry.

**Figure 6:**
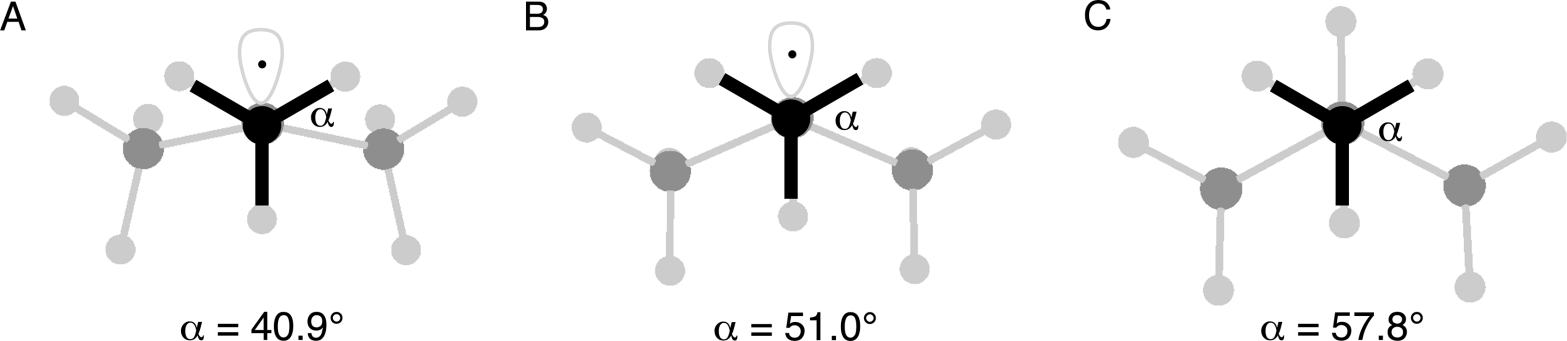
Newman-like projections down one of the C­(methyl)–C­(radical) bonds for the (A) optimized *tert*-butyl radical, (B) *tert*-butyl radical optimized with negative hyperconjugation interactions deleted, and (C) isobutane.

### Bridgehead Radicals

Bridgehead radicals are an interesting subclass of carbon-centered radicals due to the constraints placed on their adopted geometries because of their unique bicyclic structures (Figure 7).^17,19,20^ A similar set of geometry optimizations with specific hyperconjugative orbital interaction deletions was completed, as was done for the alkyl radicals discussed above. For the bridgehead radicals, the primary hyperconjugative interactions were between the SOMO and the neighboring CH bonds and (bridging) C–C bonds, as well as the opposing bridgehead CH bond (see Figure S2 in the Supporting Information for individual values). The results of these calculations are compiled in Table 4.

**Figure 7:**
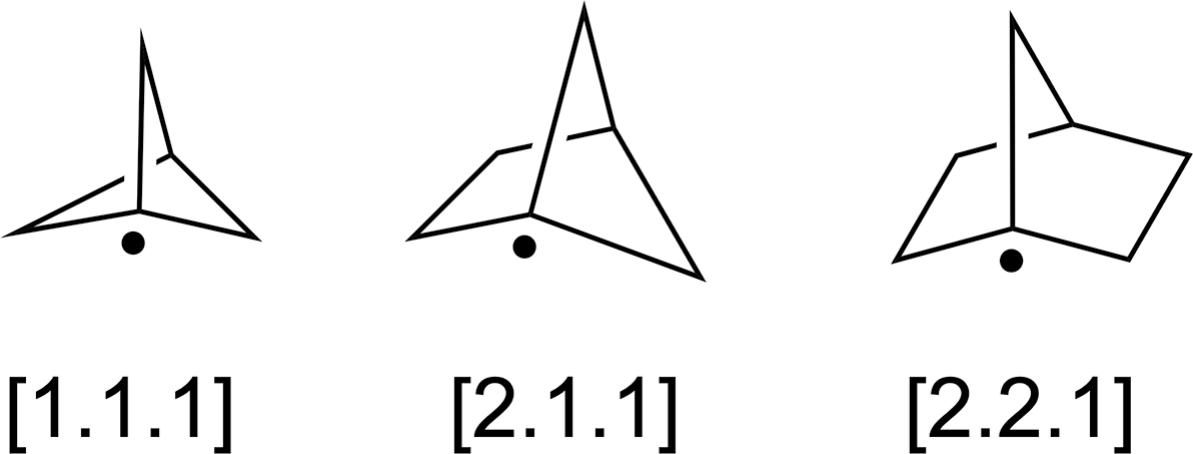
Bridgehead radicals investigated in this study.

**Table 4:** Properties of the Bridgehead Radicals from NBO Calculations on Optimized Geometries Following Specified Hyperconjugation Deletions^a^

radical	NBO deletion	ΔE (kcal/mol)	spin population (*e*)	*d* (Å)	positive hyper (kcal/mol)	negative hyper (kcal/mol)	total hyper (kcal/mol)
[1.1.1]	none	0.00	0.821	0.896	80.88	2.52	82.60
	positive	8.73	0.875	1.024	45.61	1.59	47.20
	negative	0.06	0.820	0.906	78.82	2.36	80.38
[2.1.1]	none	0.00	0.905	0.716	49.63	8.53	58.16
	positive	3.98	0.932	0.805	34.82	6.44	41.26
	negative	0.57	0.909	0.746	46.45	7.01	53.46
[2.2.1]	none	0.00	0.931	0.560	34.31	16.64	50.95
	positive	2.05	0.950	0.633	27.85	12.92	40.77
	negative	1.33	0.947	0.607	29.96	12.89	42.85

a See footnotes in Table 1 for explanations of terms.

Note that for all three bridgehead radicals, and unlike the simple alkyl radicals, deletion of the positive hyperconjugation channels had a more profound impact on the radical systems as demonstrated by the increase in spin population at the radical carbon, the extent of pyramidalization as measured by *d*, and the increase in energy of the final geometry relative to the native geometry. Furthermore, the energy contributions due to the major positive hyperconjugative stabilizations (Table 4, column 6) were substantially greater than the energies from the major negative hyperconjugation channels (Table 4, column 7). Thus, conventional hyperconjugative stabilization of the electron-poor SOMO via electron density donation from neighboring C–C bonds proved to be more important in determining the geometry of bridgehead radicals than negative hyperconjugative effects. Similar to that of the alkyl radicals, in the absence of hyperconjugative interactions, especially positive hyperconjugation, additional pyramidalization of the radical carbon centers would take place.

## Conclusions

Using the NBO-enabled technique of geometry optimizations with specific orbital interactions deleted, it is demonstrated that for simple alkyl radicals, negative hyperconjugative interactions, i.e., delocalization of the single electron in the SOMO into σ*-orbitals of aligned neighboring bonds, are predominantly responsible for their adoption of pyramidalized geometries. In the absence of these hyperconjugative interactions, torsional interactions pressure the radicals to adopt more pyramidalized geometries. For the more structurally constrained structures of bridgehead radicals, however, conventional positive hyperconjugative interactions, i.e., delocalization of electron density from σ-orbitals of aligned neighboring bonds into the electron-deficient SOMO, are especially critical to the geometries adopted. Again, in the absence of these hyperconjugative interactions, torsional interactions pressure the bridgehead radicals to adopt more pyramidalized geometries. In both cases, therefore, maximizing hyperconjugative interactions proved to be more important than minimizing torsional interactions in determining the overall radical geometry.

## Experimental Section

### Computational Methods

Geometries of all of the compounds and radicals studied were optimized using density functional theory, employing the (U)­ωB97X-D functional and the 6-311++G­(d,p) basis set using Gaussian 16W (revision B.01).^21^ Supporting Information contains the optimized geometries for all relevant structures as mol2 files, and their energies (Hartrees) are compiled in Table S1. All NBO calculations were carried out with the NBO7 program.^22^ Geometry optimizations in the presence of hyperconjugative deletions (see text) were carried out according to established protocols embedded within the NBO program.^18^ Specific hyperconjugative orbital interactions and their associated energies are provided in Figures S1 and S2 of the Supporting Information. Note that qualitatively similar energies and optimized geometry results, differing primarily only in the magnitude of second-order perturbation energies, were also obtained using the much more computationally efficient (U)­B3LYP/6-31G* method as well. NBO orbital images were rendered using Multiwfn (version 3.8).^23^ Superimposed wire structure images of radical structures were rendered using ChimeraX.^24^

## Supplementary Material





## Data Availability

The data underlying this study are available in the published article and its online Supporting Information.

## Notes

Energies of radicals following geometry optimizations with and without NBO deletions (Table S1), values for second-order perturbation energies from NBO calculations for the ethyl, 2propyl, and tert-butyl radicals (Figure S1), and values for second-order perturbation energies from NBO calculations for the [1.1.1], [2.1.1], and [2.2.1] bridgehead radicals (Figure S2) (see Experimental Section) (PDF)
Individual mol2 files for all relevant optimized structures (ZIP)

## Notes

This work is dedicated to the memory of Dr. Frederick D. Greene, former editor of this journal, a caring mentor to many, and an enthusiastic champion of physical organic chemistry.
