# Supplementary material for: What Causes Carbon-Centered Radicals to Pyramidalize? It Depends on the Type of Radical
Source: J Org Chem. 2026 Jan 5;91(2):904–10. doi: 10.1021/acs.joc.5c02141 (PMC12854734; doi:10.1021/acs.joc.5c02141)
Supplement: Supplementary file 1 [file jo5c02141_si_001.pdf]

Supporting Information

What Causes Carbon-Centered Radicals to Pyramidalize? It Depends on the Type of Radical.

Gary W. Breton*

Address: Department of Chemistry and Biochemistry, Berry College, Mount Berry, GA 30149, USA. Email: gbreton@berry.edu; Phone: 706-290-2661

Contents

Table S1. Energies of Radicals Following Geometry Optimizations With and Without NBO Deletions……………………………………………………………………………...………..…S2

Figure S1. Values for 2nd Order Perturbation Energies from NBO Calculations for the ethyl, 2-propyl, and tert-butyl radicals…………………………………………………………….....…S3

Figure S2. Values for 2nd Order Perturbation Energies from NBO Calculations for the [1.1.1], [2.1.1], and [2.2.1] bridgehead radicals…………………………….....................................…S4

Table S1. Energies of Radicals Following Geometry Optimizations With and Without NBO Deletions.

Radical	NBO Deletion[1]	Energy (Hartrees)
Methyl	none	-39.834898
Ethyl	none	-79.151338
	positive	-79.149892
	negative	-79.148706
2-Propyl	none	-118.469236
	positive	-118.466554
	negative	-118.463318
tert-Butyl	none	-157.787665
	positive	-157.783599
	negative	-157.779858
[1.1.1]	none	-194.571903
	positive	-194.557985
	negative	-194.571804
[2.1.1]	none	-233.927517
	positive	-233.921174
	negative	-233.926616
[2.2.1]	none	-273.277393
	positive	-273.274112
	negative	-273.275278

[1]none = no NBO deletions during geometry optimization; positive = major positive hyperconjugative channels deleted during geometry optimization; negative = major negative hyperconjugative channels deleted during geometry optimization.

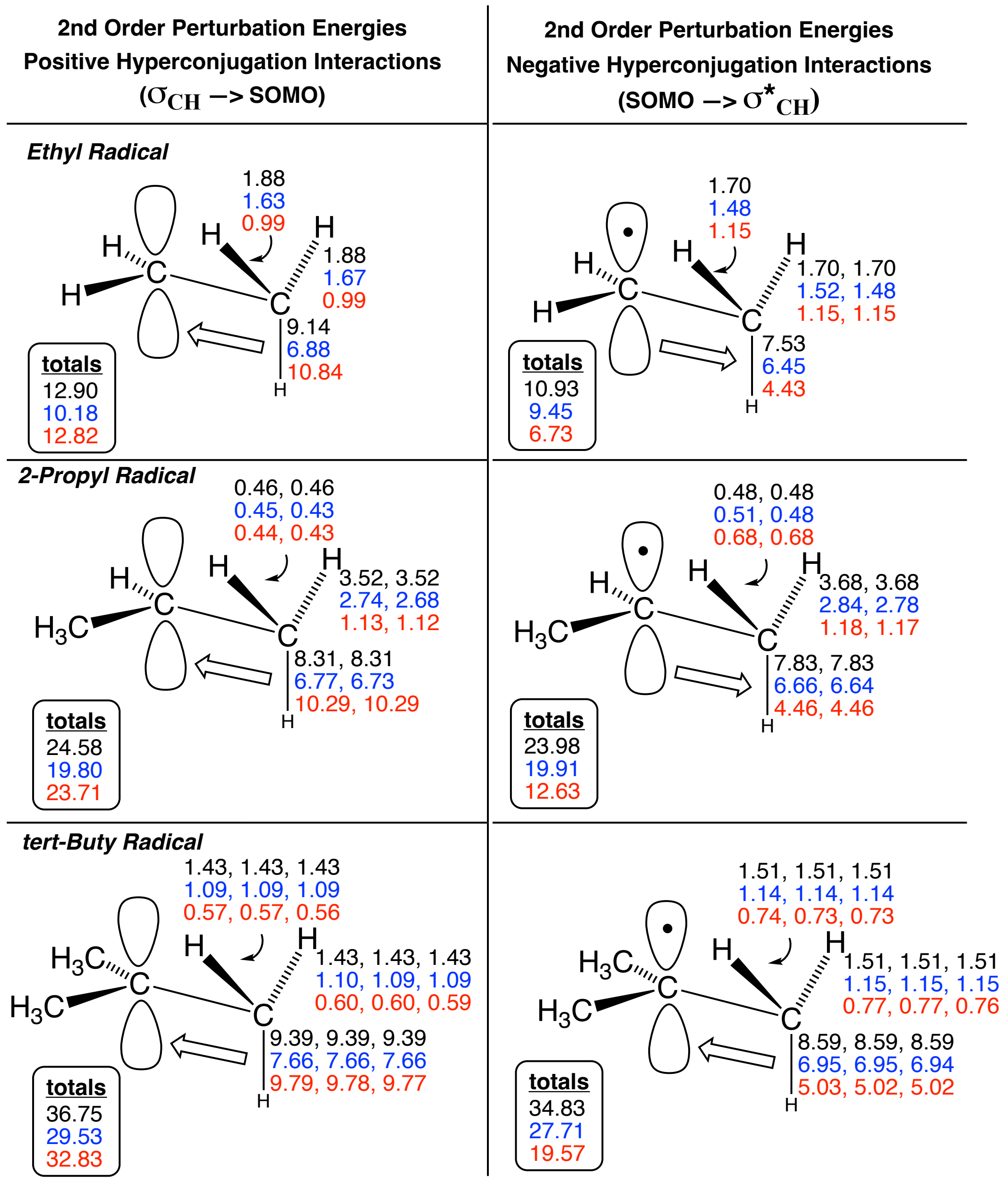

Figure S1. Values (kcal/mol) for 2nd order perturbation energies from NBO calculations correlating with i) positive hyperconjugation interactions (left column) and negative hyperconjugation interactions (right column) for the ethyl, 2-propyl, and *tert*-butyl radicals. The figures in black are the energies in the absence of any deletions. The figures in blue are energies from the structures optimized with positive hyperconjugation channel deletions. The figures in red are energies from the structures optimized with negative hyperconjugation channel deletions. Multiple values on the same line correlate with position-related CH bonds on the other methyl group(s). The "totals" values correlate with the energies reported in Tables 1-3.

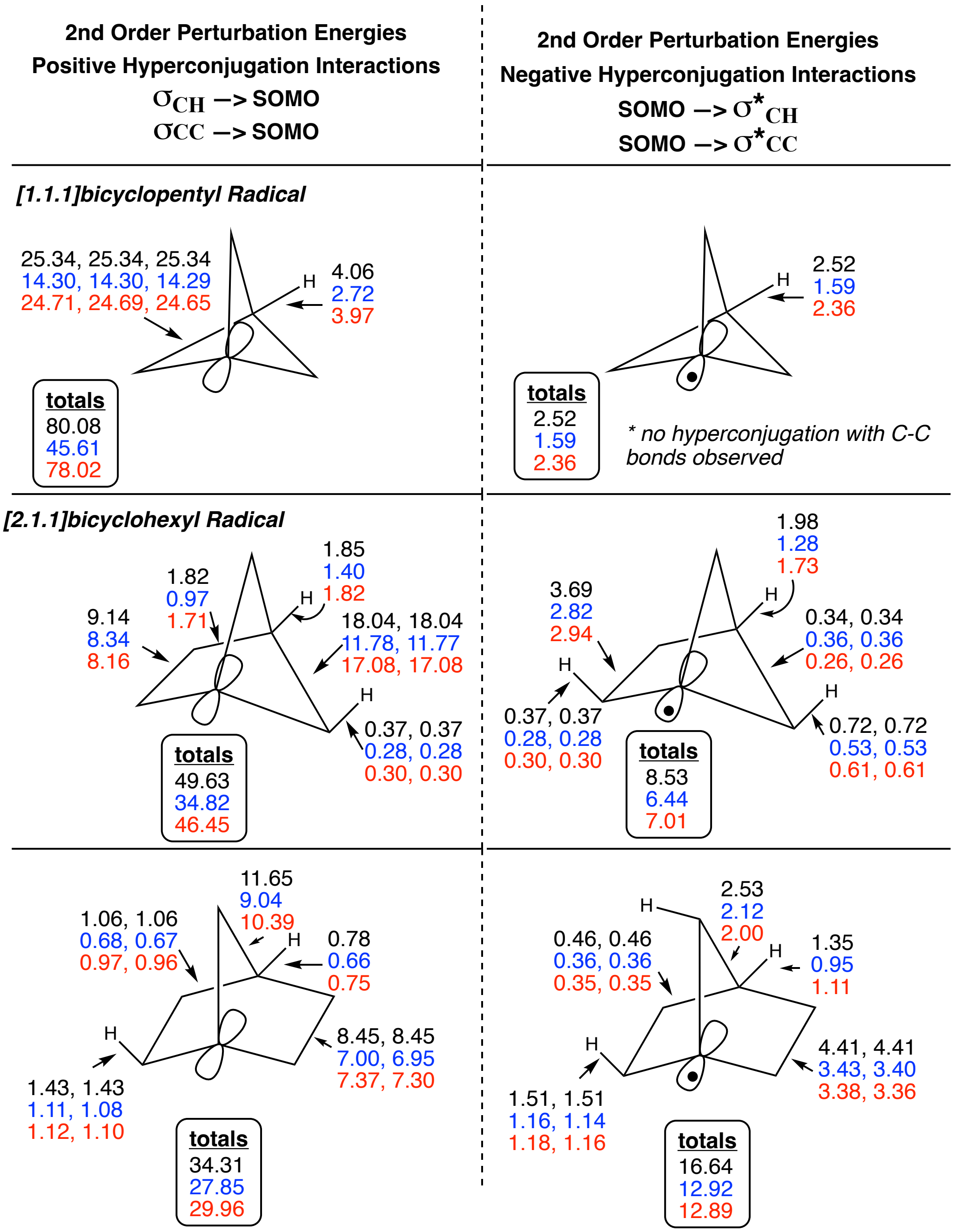

Figure S2. Values (kcal/mol) for 2nd order perturbation energies from NBO calculations correlating with i) positive hyperconjugation interactions (left column) and negative hyperconjugation interactions (right column) for the [1.1.1], [2.1.1], and [2.2.1] bridgehead radicals. The figures in black are the energies in the absence of any deletions. The figures in blue are energies from the structures optimized with positive hyperconjugation channel deletions. The figures in red are energies from the structures optimized with negative hyperconjugation channel deletions. Multiple values on the same line correlate with position-related CH bonds on the other methyl group(s). The "totals" values correlate with the energies reported in Table 4.
